# Breaking Down the Pain Pathway: Bacterial Proteases Activate Nociceptors to Cause Pain

**DOI:** 10.1016/j.jcmgh.2024.03.009

**Published:** 2024-04-05

**Authors:** Christophe Altier

**Affiliations:** Department of Physiology and Pharmacology, Cumming School of Medicine, University of Calgary, Calgary, Alberta, Canada; Inflammation Research Network-Snyder Institute for Chronic Diseases, Cumming School of Medicine, University of Calgary, Calgary, Alberta, Canada; Alberta Children's Hospital Research Institute, University of Calgary, Calgary, Alberta, Canada; Hotchkiss Brain Institute, Cumming School of Medicine, University of Calgary, Calgary, Alberta, Canada

The recognition of the importance of the microbial ecosystem in physiology and drug sensitivity has led to an exciting era in biomedical research. The microbiome-gut-brain axis involves a bidirectional communication that shapes neurodevelopment and neural circuits,^1^ modulating behaviors, sensory functions, and contributing to psychiatric disorders and neurologic diseases.^2^ Perturbations in the microbial balance within the gastrointestinal tract stand out as a key driver of abdominal pain, triggered by such factors as inflammation, diet, antibiotic treatments, and even stress. Understanding the biologic mechanisms of dysbiosis-induced pain is crucial to get insight into the cause of pain in such conditions as inflammatory bowel disease and irritable bowel syndrome,^3^ paving the way for the discovery of potential pain biomarkers and safer treatments for pain management.

Pain symptoms are mediated by a specialized subset of sensory neurons known as nociceptors, finely tuned to detect noxious physical and chemical stimuli. Nociceptors densely innervate the skin and internal organs, including the colonic mucosa, where they interact with epithelial and immune cells while sensing microbial-derived metabolites and toxins.^4^ Despite this intricate network, the precise mechanisms through which dysbiosis activates nociceptors to initiate pain sensations remain elusive.

Proteases constitute an important class of enzymes that can directly or indirectly activate nociceptors at epithelial and mucosal barriers.^5^ The activation of protease-activated receptor 2 (PAR2), located at the nerve endings of the nociceptors, has long been implicated in inflammatory, visceral pain, and cancer-evoked pain, whereas PAR1 evokes itch responses in the skin.^6^ PAR2 activation induces sensitization of nociceptive ion channels (TRPV1, TRPA1, VGCC channels) and long-lasting neuronal plasticity that are dependent on the ERK and a BDNF/TrkB/PKC signaling axis, leading to a chronic pain state.^7^

In this issue of *Cellular and Molecular Gastroenterology and Hepatology*, Baker et al^8^ examined the mechanisms by which antibiotic-induced dysbiosis triggers hypersensitivity within visceral and somatic pain pathways. Their findings reveal that vancomycin induces visceral hypersensitivity in response to colorectal distention, and thermal hypersensitivity to noxious heat. Through electrophysiologic recordings, they demonstrate that cultured dorsal root ganglion neurons from vancomycin-treated mice exhibit hyperexcitability. Moreover, treating control dorsal root ganglion neurons with serum from vancomycin-treated mice enhances neuronal activity. Using pharmacologic tools and PAR2-deficient nociceptors, they found that dysbiosis mediates neuronal hyperexcitability via cysteine proteases acting on the PAR2 receptor.

The study provides important information on the mechanisms of dysbiosis-induced pain, suggesting that antibiotic-induced dysbiosis mediates the production of bacterially derived proteases. Accordingly, PAR2 activation by proteases is a well-described process of hyperexcitability and pain in visceral organs and the skin. Moreover, the findings indicate that pronociceptive proteases produced by the dysbiotic microbiome can act locally and systematically on PAR receptors to elicit pain.

Another interesting family of receptors enriched in nociceptors is the Mas-related GPCR family (Mrgpr). These receptors respond to several pharmacologic tools for PAR2. In vivo studies using nociceptor-specific knockout of PAR2 will further support Baker et al’s^8^ electrophysiologic observations and address the contribution of Mrgpr found on nociceptors or mucosal mast cells. Furthermore, the study by Baker et al^8^ reports that antibiotic-induced dysbiosis induced thermal hyperalgesia, whereas direct activation of PAR2 in the skin was shown to evoke mechanical pain. Considering that PAR2 is expressed in a discrete and unique population of dorsal root ganglion neurons (Nppb+; Il31Ra+) known for its role in itch and pain,^9^ further work using genetically engineered mouse lines for conditional knockout of PAR2 in these neurons will be valuable to determine whether one or more subsets of sensory neurons respond to dysbiosis to elicit pain.

Finally, as indicated in the study, commensal bacteria, such as *Lactobacillus paracasei*, *Lactobacillus reuteri*, and *Faecalibacterium prausnitzii*, exhibit opposite effects, suppressing the excitation of nociceptors. Exploring whether these microbial-derived antinociceptive effects are mediated by antiproteases or other metabolites, along with their mechanisms of action, will be intriguing avenues for further investigation.

Microbial therapeutics is a growing field of research with applications in a wide range of conditions, including gastrointestinal and neurologic disorders, and allergies. The study by Baker et al^8^ supports the notion that restoring the microbial balance may be beneficial for several pain conditions triggered or exacerbated by bacterial-derived metabolites. Lastly, the critical period of gut microbiota colonization coincides with a pivotal postnatal phase during which nociceptors acquire their molecular identity and specificity. Therefore, early life microbial perturbations, in response to environmental factors, infection, or antibiotic use, may influence the development of chronic pain conditions later in life.
